# Supplementary material for: Deletion of wfs1 Impairs Oligodendrocyte Precursor Cells Dorsal Distribution and Myelination Through the wfs1-hmgcs1 Axis in Zebrafish
Source: Biology (Basel). 2026 Aug 16;15(16):1402. doi: 10.3390/biology15161402 (PMC13510151; doi:10.3390/biology15161402)
Supplement: Supplementary file 1 [file biology-15-01402-s001.zip › File S1-Transcriptome sequencing data and differentially expressed genes (DEGs) in wfs1-- zebrafish at 5 dpf/2.KEGG/wfs1KOvsWT/UP/src/dre00590.html]

dre00590


- dre:393592

- Up regulated genes

393592(3.73058515777409)

- dre:393592

- Up regulated genes

393592(3.73058515777409)

- dre:393592

- Up regulated genes

393592(3.73058515777409)

- dre:393592

- Up regulated genes

393592(3.73058515777409)

- dre:393592

- Up regulated genes

393592(3.73058515777409)

- dre:393592

- Up regulated genes

393592(3.73058515777409)

- dre:393592

- Up regulated genes

393592(3.73058515777409)

- dre:393592

- Up regulated genes

393592(3.73058515777409)

- dre:559020

- Up regulated genes

559020(1.27287729194892)

- dre:406370

- Up regulated genes

406370(7.33922178722892)
- dre:560649

- Up regulated genes

560649(1.22362655394035)

- dre:406575

- Up regulated genes

406575\_1(1.68114903804516)

- dre:559020

- Up regulated genes

559020(1.27287729194892)

- dre:406449

- Up regulated genes

406449\_1(6.49480297810026)

- dre:393297

- Up regulated genes

393297(1.19337638849417)

- dre:393592

- Up regulated genes

393592(3.73058515777409)

- dre:393297

- Up regulated genes

393297(1.19337638849417)

- dre:494108

- Up regulated genes

494108(2.34015565356837)
- dre:566996

- Up regulated genes

566996(3.78724584427087)

- dre:494108

- Up regulated genes

494108(2.34015565356837)
- dre:566996

- Up regulated genes

566996(3.78724584427087)

Close
